# Constitutive Expression of miR408 Improves Biomass and Seed Yield in Arabidopsis

**DOI:** 10.3389/fpls.2017.02114

**Published:** 2018-01-25

**Authors:** Zhaoqing Song, Lifen Zhang, Yulong Wang, Haixia Li, Shuang Li, Huijie Zhao, Huiyong Zhang

**Affiliations:** State Key Laboratory of Wheat and Maize Crop Science, Collaborative Innovation Center of Henan Grain Crops, College of Life Science, Henan Agricultural University, Zhengzhou, China

**Keywords:** miR408, biomass, seed yield, photosynthesis, copper

## Abstract

miR408 is highly conserved among different plant species and targets transcripts encoding copper-binding proteins. The function of miR408 in reproductive development remains largely unclear despite it being known to play important roles during vegetative development in Arabidopsis. Here, we show that transgenic Arabidopsis plants overexpressing *MIR408* have altered morphology including significantly increased leaf area, petiole length, plant height, flower size, and silique length, resulting in enhanced biomass and seed yield. The increase in plant size was primarily due to cell expansion rather than cell proliferation, and was consistent with higher levels of myosin gene expression and gibberellic acid (GA) measured in transgenic plants. In addition, photosynthetic rate was significantly increased in the MIR408-overexpressing plants, as manifested by higher levels of chloroplastic copper content and plastocyanin (PC) expression. In contrast, overexpression of miR408-regulated targets, *Plantacyanin* and *Laccase 13*, resulted in reduced biomass production and seed yield. RNA-sequencing revealed that genes involved in primary metabolism and stress response were preferentially enriched in the genes upregulated in *MIR408*-overexpressing plants. These results indicate that miR408 plays an important role in regulating biomass and seed yield and that *MIR408* may be a potential candidate gene involved in the domestication of agricultural crops.

## Introduction

miRNAs are a class of fundamental, sequence-specific regulatory small RNA molecules that repress target gene expression post-transcriptionally. In plants, these 20–24 nucleotide-long RNA species are processed from stem-loop structured precursors by a Dicer-like enzyme and are integrated into silencing complexes, where, in general, they function as gene repressors by directing cleavage of complementary mRNA transcripts (Llave et al., 2002; Brodersen et al., 2008; Voinnet, 2009; Rogers and Chen, 2013). Additionally, miRNAs can also mediate DNA methylation and histone modification to affect gene expression (Schramke and Allshire, 2004; Wu et al., 2010; Khraiwesh et al., 2012).

As regulatory molecules, miRNAs share a common regulatory logic with transcription factors (Chen and Rajewsky, 2007; Hobert, 2008). For instance, miRNAs recognize their target mRNAs based on short complementary sequence. A single miRNA can potentially regulate the expression of multiple target genes, and multiple miRNAs may act synergistically to regulate the same genes. This, together with the large number of miRNA genes, indicates that miRNAs may have a substantial impact on the transcriptome post-transcriptionally. Accumulating evidence has shown that miRNAs are involved in the control of almost all biological and metabolic processes in plants. A number of these processes are potentially responsible for some of the most challenging plant traits in agricultural production, such as regulation of plant development and plant architecture, and responses to environmental stresses and defense (Jones-Rhoades et al., 2006; Garcia and Frampton, 2008; Rubio-Somoza and Weigel, 2011; Comai and Zhang, 2012; Khraiwesh et al., 2012; Sun, 2012). Thus, advances in functional identification of miRNAs and miRNA networks will open up new avenues for understanding the genetic mechanisms underlying these complex traits (Sun, 2012). Moreover, it is believed that miRNAs themselves can be regarded as a reservoir of resourceful genes for modifying these challenging traits in agricultural production (Sun, 2012; Zhou et al., 2013).

MiR408 is one of the most conserved miRNA families and has, to date, been annotated in more than 30 plant species (Axtell and Bowman, 2008; Kozomara and Griffiths-Jones, 2011), implying that its function is fundamental to plants. It has been reported in various plant species that miR408 is differentially expressed in response to a variety of environmental cues, including copper, light, mechanical stress, dehydration, cold, and reactive oxygen species (Lu et al., 2005; Yamasaki et al., 2007; Abdel-Ghany and Pilon, 2008; Kantar et al., 2010; Li et al., 2010; Trindade et al., 2010; Mutum et al., 2013; Zhang and Li, 2013; Zhang et al., 2014; Ma et al., 2015). Moreover, Feng et al. (2013) reported that tae-miR408 was positively correlated with the resistance of host plants to abiotic stresses and stripe rust by regulating one of its target genes, chemocyanin-like protein gene (*TaCLP1*), in wheat. Thus, these observations indicate that miR408 is a key regulatory hub in abiotic and biotic stress signaling. Expression of *MIR408* gene adapting to the diverse environmental stresses suggests that multiple different transcription factors may be involved in its regulation. However, our knowledge regarding the regulation of *MIR408* is still relatively sparse, and just a few of transcription factors, known as LONG HYPOCOTYL 5 (HY5) and SQUAMOSA PROMOTER-BINDING PROTEIN-LIKE 7 (SPL7), have been reported to directly regulate *MIR408* transcription in response to varying light and copper conditions (Yamasaki et al., 2009; Zhang and Li, 2013; Zhang et al., 2014).

Previous studies have indicated that miR408 targets several genes for copper-binding proteins that belong to two distinct families of phytocyanin and laccase. Plastocyanin is a copper-binding protein and functions as a mobile electron carrier between the membrane-bound cytochrome *b6f* complex and P-700, the reaction center of photosystem I (PSI) in eukaryotic photosynthetic organisms (Raven et al., 1999; Joliot and Joliot, 2006). In Arabidopsis, there have two plastocyanin genes (*PETE1* and *PETE2*), and *PETE2* is the predominant isoform. Plants with mutations in the plastocyanin genes show impaired growth (Abdel-Ghany, 2009; Pesaresi et al., 2009). Laccases are also copper-containing oxidase enzymes and play a role in the formation of lignin by promoting the oxidative coupling of monolignols, such as LAC3, LAC4, LAC 12, and LAC13 (Zhao et al., 2013; Schuetz et al., 2014; Wang et al., 2014). Copper (Cu) plays a vital role in plant growth and development because it acts as a cofactor of many proteins and enzymes involved in number of physiological processes, such as photosynthesis, seed production, carbohydrate distribution, nitrogen fixation, antioxidant activity, cell wall metabolism and hormone perception (Pilon et al., 2006). The Cu-microRNAs are responsible for distribution of Cu allowing plants to coordinate Cu protein expression and properly development (Pilon, 2017). Taken together, these findings clearly demonstrate the importance of miR408 in copper homeostasis.

Our previous studies have revealed that miR408 accumulation promotes vegetative growth as well as biosynthesis of pigments in Arabidopsis young seedlings (Zhang et al., 2011, 2014; Zhang and Li, 2013). However, the underlying molecular mechanisms remain unclear. More importantly, it is not yet clear how miR408 affects reproductive development. Dong et al. (2005) demonstrated that overexpression of *plantacyanin* (*ARPN*) led to reduction of seed production in Arabidopsis. More recently, Zhao et al. (2016) reported that overexpression of tae-miR408 promoted heading time as well as increased transgenic plant height in wheat. These observations indicate that miR408 may play a role in reproduction in plants. To develop a better understanding of the biological function of miR408, further physiological characterization of miR408 transgenic plants and functional analysis of its targets at the molecular level become essential. In this work, we present evidence supporting the involvement of miR408 in different growth stages, further illustrating its functional regulatory role in biomass production and seed yield.

## Materials and Methods

### Plant Materials and Culture Conditions

Wild-type plants used were the *Arabidopsis thaliana* ecotype Columbia-0. The *35S:MIR408* (*MIR408-OX*) and *amiR408* lines were previously described by Zhang and Li (2013). The T-DNA insertion lines of SALK_091945 for *ARPN* and SALK_023935 for *LAC13* were from the Arabidopsis Biological Resource Center (ABRC). The sterilized seeds were sown on half-strength Murashige and Skoog (MS) medium after storage at 4°C for 4 days. The seedlings were grown in a growth chamber with the following settings: standard long-day (16 h of light/8 h of darkness) conditions at 23 ± 1°C, light intensity of 100–120 μmol m^-2^ s^-1^, and a relative humidity of approximately 50%. All phenotypic characterization experiments were conducted on multiple biological samples and repeated at least three times.

### Generation of Transgenic Plants

To obtain the *35S:Plantacyanin* (*ARPN-OX*) transgenic plants, the full length cDNA of *Plantacyanin* gene was amplified by PCR using primer pairs with restriction endonuclease cleavage site, and inserted into the pJim19 binary vector (Basta resistance; Zhang et al., 2008), then transformed into the wild type background. Transgenic plants were selected with 20 mg/L Basta. T3 generation homozygous lines were used for all experiments.

To obtain the *35S:LAC13* (*LAC13-OX*) transgenic plants, the full length cDNA of *LAC13* was amplified by PCR using primer pairs with restriction endonuclease cleavage site, and inserted into the pJim19 binary vector (Hygromycin resistance; Zhang et al., 2008), then transformed into the wild type background. Transgenic plants were selected with 40 mg/L hygromycin. T3 generation homozygous lines were used for all experiments.

The *ARPN-OX*/*LAC13-OX* plants were generated by crossing *ARPN-OX* and *LAC13-OX* transgenic plants. F2 or F3 homozygous progenies were identified by growing the seedlings on MS medium containing both hygromycin and basta.

All the PCR products were obtained using Phusion high-fidelity DNA polymerase (NEB^1^) and the resultant constructs were sequenced by GENWIZE to ensure their integrity. The plasmids were electroporated into *Agrobacterium tumefaciens* GV3101, respectively. Arabidopsis transformation was performed by the floral dip method (Clough and Bent, 1998). All of the primers used to generate the above-mentioned constructs are listed in Supplementary Table S2.

### RNA Analyses

Total RNA was extracted from Arabidopsis seedlings using the RNeasy plant mini kit (Qiagen) and quantified by NanoDrop 2000, and the integrity was examined by agarose gel electrophoresis. For real-time qRT-PCR, 5 μg of DNaseI treated RNA was reverse transcribed using the SuperScript II reverse transcriptase (Invitrogen), and the resultant cDNA was analyzed using the Power SYBR Green PCR Master Mix (Takara) with a Bio-Rad CFX96 real-time PCR detection system in triplicate. A total volume of 20 μl reaction system was set up according to the manufacture’s instruction, including 10 μl of 2x SYBR Green Master Mix, 0.2 μM of forward primer, 0.2 μM of reverse primer, 50 ng of cDNA template, ddH_2_O up to 20 μl. The *Actin2* amplicon was used for normalization. Determination of relative gene expression level was carried out using the standard 2^-ΔΔ*C*(T)^ method. All experiments were performed on three independent biological samples with each including three technical replicates. Statistical significance was calculated using the two-tailed Student’s *t*-test. For RNA gel blot analysis, 20 μg of total RNA were loaded per lane and blotting was performed as described previously. Fragments of *ARPN* and *LAC13* used for probe labeling were generated by PCR. Primer sequences are listed in Supplementary Table S2.

### Glucose Measurement

Measurement of glucose content was performed using the Glucose and Sucrose Assay Kit (Biovision) according to the manufacturer’s instructions as described previously (Zhang et al., 2014). Rosette leaves from the plants grown in soil under standard long-day (16 h of light/8 h of darkness) conditions for 2 weeks were homogenized in a glucose assay buffer, and the supernatant was collected after centrifuging at 12,000 rpm for 10 min. The reaction system was set up in a 100-μL total volume including 50 μL of sample in glucose assay buffer and 50 μL of glucose assay mix (46 μL of glucose assay buffer, 2 μL of glucose probe, and 2 μL of glucose enzyme mix) and incubated at 37°C for 30 min. The *A*_570_ was collected, and glucose concentrations of the test samples were calculated based on the standard curve. The experiments were performed on three independent biological samples.

### Immunoblotting

Proteins were extracted from the rosette leaves of plants grown in soil under standard long-day (16 h of light/8 h of darkness) conditions for 2 weeks, and immunoblotting was performed as described previously (Feng et al., 2004). After electroblotting on a nitrocellulose membrane, protein gel blot analysis was performed using antibodies against plastocyanin (Acris Antibodies, AS06141). Detection was performed using goat anti-rabbit IgG (H+L) horseradish peroxidase conjugate secondary antibodies (Sigma). RPT5 (Abcam, ab22676) was used as loading control.

### Dry Weight and Cell Size Measurements

Dry weight was measured by drying aerial parts of 2-week-old plants grown in soil at 80°C for 10 h. For cell size measurements, a fully expanded first rosette leaf was fixed in FAA [formalin:glacial acetic acid:ethanol (70%) = 1:1:18)]. The epidermal cells of the leaves or the petioles were peeled off by hand, and then photographed using a Leica DM5500 microscope. The epidermal cell area and mesophyll cell length were measured using ImageJ analysis software.

### RNA Sequencing

Total RNA from three biological replicates was extracted with an RNeasy plant mini kit (Qiagen) from the aerial parts of 10-day-old plants grown on MS plate under continuous light condition. Library construction and sequencing on the HiSeq 2000 platform were performed according to the manufacturer’s instructions (Illumina) by BioMarker cooperation (Beijing, China). Bowtie (Langmead et al., 2009) was used to map the sequence reads to the Arabidopsis genome (TAIR 10) and only uniquely mapped reads were used in subsequent analyses. Reads mapped to exonic regions of annotated gene models were normalized against the length of the transcript. On average, 74.4% of the total reads mapped to the Arabidopsis reference genome sequence (Supplementary Table S3), with most of the reads mapping to exons and only a small portion mapping to introns or intergenic regions. To compare gene expression between wild type and *MIR408-OX* genotypes, length-normalized read density was quantile-normalized. Relevant genes were those identified as differing in expression by at least 1.5-fold (*p* < 0.01) in the test and control samples. The raw sequence data reported in this paper have been deposited in the Genome Sequence Archive (Wang et al., 2017a) in BIG Data Center (Wang et al., 2017b), Beijing Institute of Genomics (BIG), Chinese Academy of Sciences, under accession number CRA000553 that are publicly accessible at http://bigd.big.ac.cn/gsa.

### Measurement of Photosynthesis and Contents of Cellular Copper and Gibberellins

The net photosynthesis was measured using a portable photosynthesis system (CIRAS-1, PP Systems, United Kingdom) by an open system. Measurements were made by attaching a light source to the leaf chamber window under saturating photosynthetic photon flux densities (1500 μmol m^-2^ s^-1^), ambient CO2 concentration (Ca) at 290 μmol mol^-1^, and relative humidity of 50–60%. Leaf temperature and vapor pressure deficit (VPD) were maintained at 28°C and 0.99 ± 0.2 kPa, respectively. Data were determined at least in six leaves from six different plants of each genotype grown in soil for 2 weeks in a growth chamber.

For cellular copper content analysis, 10-day-old seedlings grown on half MS medium under continuous light were harvested and weighted, and then washed twice with 1 mM EDTA and once with double-distilled water. The following treatment and measurement were performed as previously described (Zhang et al., 2014).

The endogenous hormone content was measured using the Agilent 1100 high-performance liquid chromatography (HPLC) system (Agilent, Palo Alto, CA, United States). The aerial parts from more than fifteen individual plants of wild type and *MIR408-OX* plants grown in soil for 2 weeks were cold-dried under vacuum. 0.2 g dried leaves were added to 10 mL 80% aqueous methanol and immediately homogenized on ice, then kept at 4°C overnight in darkness with continuous shaking. The homogenates were centrifuged for 10 min at 4500 *g* at 4°C, and the supernatant was collected and evaporated under vacuum. Dry residue was re-dissolved in 5 mL of ammonium acetic buffer (0.1 M, pH 9.0) and centrifuged at 14 000 rpm for 20 min, and the supernatant was collected and purified sequentially through Polyvinylpolypyrrolidone (PVPP) column and DEAE Sephadex A225 column. Before HPLC analysis, the elution with 50% aqueous methanol was concentrated by Sep-Pak C18 column (Waters Chromatography). Standard gibberellins were purchased from Fluka Co. (Switzerland), and all solvents and buffers were HPLC-quality. Two biologically independent replicates were performed.

## Results

### Phenotypic Effects of Increased miR408 Expression in Arabidopsis

**FIGURE 1:**
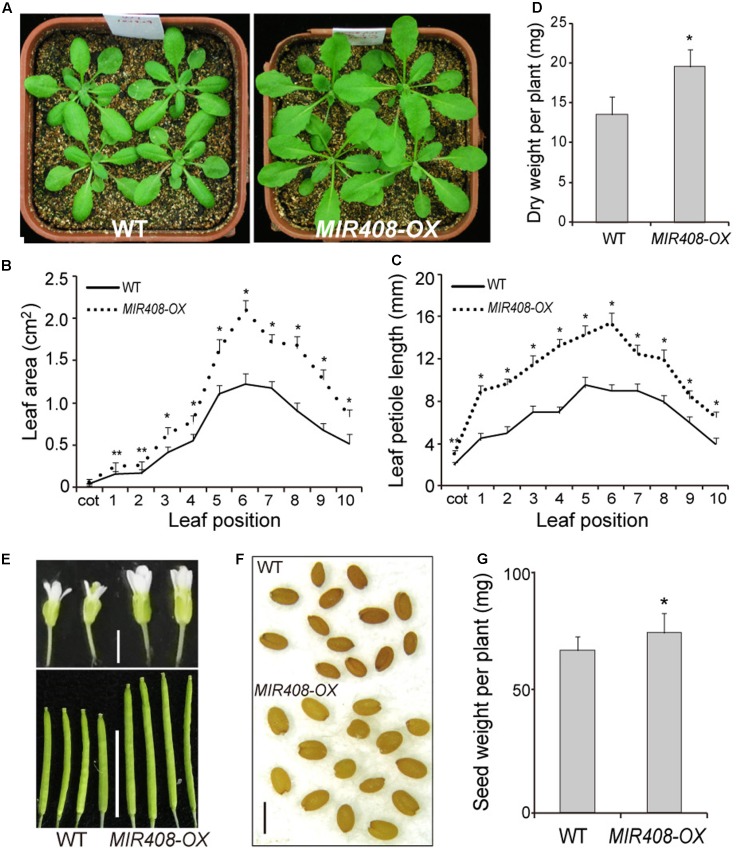
Effects of miR408 overexpression on Arabidopsis development. **(A)** Wild type (left) and miR408-overexpressing plants (*MIR408-OX*; right) grown in soil for 2 weeks. **(B,C)** Leaf area **(B)** petiole length **(C)** of individual leaves of the wild type and *MIR408-OX* plants grown in soil for 2 weeks (*n* = 15). **(D)** Dry weight was determined for 2-week-old plants grown in soil (*n* = 20). **(E)** Comparison of flowers (upper, scale bar = 2 mm) and silique (lower, scale bar = 1 cm) between the wild type and *MIR408-OX* plants. **(F)** Seed morphology of the wild type and *MIR408-OX* plants, scale bar = 500 μm. **(G)** Seeds from more than 15 plants were collected and quantified. Data are means ± SD from *n* biological repeats. ^∗^*p* < 0.01, ^∗∗^*p* < 0.05 vs. wild type, by two-tailed Student’s *t*-test.

Constitutive expression of miR408 in Arabidopsis implies that it has the potential to act throughout the growth and development, and its importance at the seedling stage is well known (Zhang and Li, 2013). To determine the phenotypic impact of increased miR408 expression during the later development, transgenic plants overexpressing miR408 (*MIR408-OX*; Zhang and Li, 2013) were examined. The *MIR408-OX* transgenic plants grew rapidly and were morphologically larger than wild type (**Figure 1A**). In plants grown in soil for 2 weeks after stratification, the areas of rosette leaves of *MIR408-OX* transgenic plants were significantly increased compared with the ones of wild type (**Figure 1B**). The average petiole length from the cotyledon to the tenth leaves of the *MIR408-OX* transgenic plants also becomes much longer than that of wild type (**Figure 1C** and Supplementary Figures S1a,b). In addition, both fresh weight and dry weight of the *MIR408-OX* plants were dramatically increased compared with wild type (Supplementary Figure S1c and **Figure 1D**). While grown in soil for 6 weeks, the transgenic plants were much taller than wild type (Supplementary Figure S1d). In the plants at the reproductive stage, a comparison of flower size and silique length indicated significant increase for the *MIR408-OX* transgenic plants (**Figure 1E** and Supplementary Figure S1e). Furthermore, wild type plants exhibited a darker seed coat color than the transgenic ones (**Figure 1F**), and the seed yield of the *MIR408-OX* plants significantly increased (**Figure 1G**). In summary, the data show that constitutive expression of miR408 in wild type background leads to changes in morphology as well as increases in biomass and seed yield.

### Cell Expansion and Elongation Likely Contributes to the Larger Phenotypes

**FIGURE 2:**
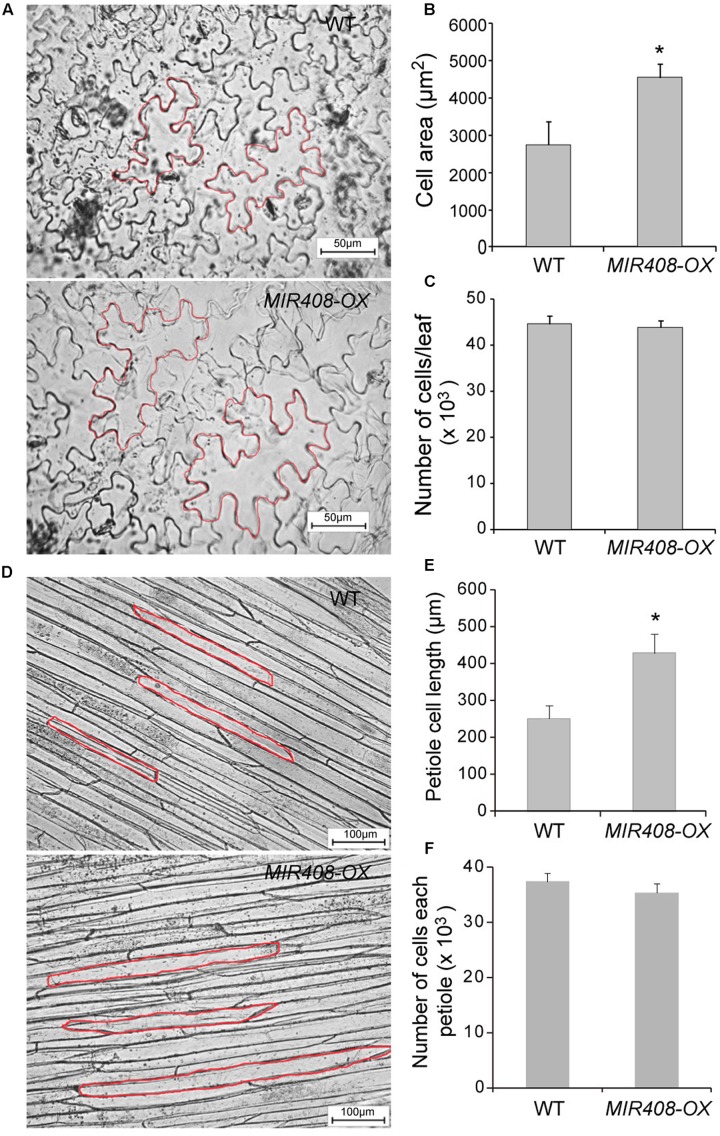
Cell size and number. **(A)** Epidermal cells of the first rosette leaf from the wild type (upper) and *MIR408-OX* plants (lower). The scale bars represent 50 μm. **(B)** Epidermal cell area of the first rosette leaf (*n* = 50). **(C)** The number of cells per leaf (*n* = 10). **(D)** Epidermal cells of the first leaf petiole from the wild type (upper) and *MIR408-OX* (lower) plants. The scale bars represent 50 μm. **(E)** Cell length of the first leaf petiole epidermal cell (*n* = 50). **(F)** The number of cell from the first leaf petiole (*n* = 10). Data are means ± SD from n biological repeats. ^∗^*p* < 0.01 vs. the wild type, by two-tailed Student’s *t*-test.

Either increased cell number or cell length could lead to larger leaf size (Tsukaya et al., 2002). To investigate whether enhanced leaf area in the *MIR408-OX* plants was primarily a result of cell division or cell expansion, the lengths of cells in the middle of the first rosette leaf were measured. Microscopic analysis revealed that the epidermal cell size of the transgenic plants was about 165% that of wild type (**Figures 2A,B**). However, the total number of epidermal cells showed no obvious changes in these two genotypes (**Figure 2C**). Consistent with the markedly enhanced leaf petiole elongation, the dramatic increase in the epidermal cell length was observed in the transgenic plants (**Figures 2D,E**) meanwhile the cell number showed no difference between wild type and *MIR408-OX* transgenic plants (**Figure 2F**). Together, increased leaf size was in good agreement with the increase in the epidermal cell length, suggesting that enhanced leaf size or leaf petiole elongation in the *MIR408-OX* plants primarily resulted from an increase in cell expansion rather than cell proliferation.

### Enhanced Expression of Genes Involved in Cytoplastic Growth and GA Biosynthesis in the *MIR408-OX* Plants

**FIGURE 3:**
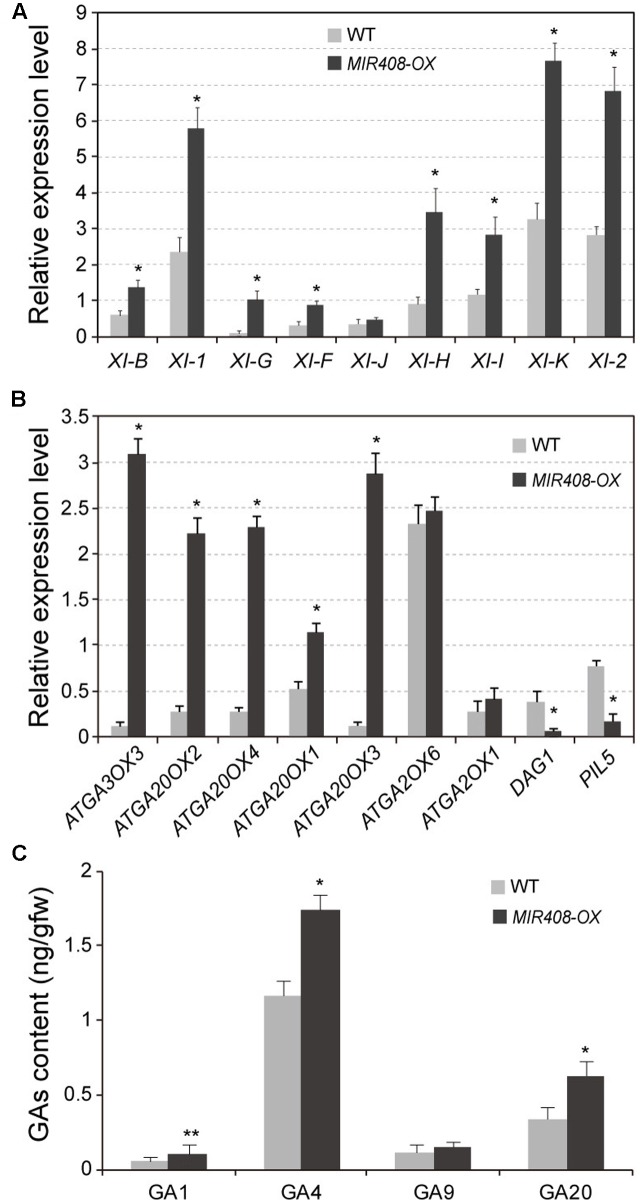
Expression levels of myosin and GA biosynthetic genes in the wild type and *MIR408-OX* transgenic plants. **(A,B)** Quantitative RT-PCR analysis of mRNA levels for myosin genes **(A)** and GA biosynthetic genes **(B)** using the rosette leaves from the plants grown in soil for 2 weeks. *Actin2* was used as an internal control. Data are means ± SD of three biological experiments. **(C)** Levels of endogenous GAs in wild type and *MIR408-OX* plants. Data are means ± SD of three technical repeats. ^∗^*p* < 0.01, ^∗∗^*p* < 0.05 vs. the wild type, by two-tailed Student’s *t*-test.

Previous reports indicated that cytoplasmic streaming, a key determinant of plant size, is generated by organelle associated myosin XI moving (Shimmen and Yokota, 2004; Ojangu et al., 2012; Tominaga et al., 2013). Gene knockout analyses of several myosin XI members lead to growth defects concomitant with reduction in cell size (Prokhnevsky et al., 2008; Peremyslov et al., 2010; Ojangu et al., 2012). Therefore, we examined the expression of nine myosin XI genes, including *XI-B, XI-1, XI-G, XI-F, XI-J, XI-H, XI-I, XI-K, and XI-2*. Compared to wild type, eight of the tested genes except for *XI-J* exhibited dramatically increased transcript levels in the *MIR408-OX* transgenic plants (**Figure 3A**). Among these myosin members, *XI-K* and *XI-2* transcripts were the most abundant, which is consistent with the conclusion that *XI-2* and *XI-K* are considered the major myosins providing the motive force for cytoplasmic streaming (Peremyslov et al., 2008; Ueda et al., 2010). Thus, it is likely that high level accumulation of myosin in the *MIR408-OX* transgenic plants might drive cytoplasmic growth, thereby promoting cell expansion and thus leading to the larger morphology.

Previous studies showed that alteration of Gibberellin (GA) metabolism or disruption of the GA signal transduction plays a critical role in regulation of plant dwarfism (Hedden and Phillips, 2000; Monna et al., 2002; Sasaki et al., 2002). Overexpression or repression of genes for enzymes in GA biosynthesis could lead to the alteration of GA levels and thereby result in dwarf or tall phenotypes. Thus, we determined the expression levels of genes involved in GA biosynthesis. As shown in **Figure 3B**, the transcript levels of biosynthetic genes for GA20-oxdases (GA20OX) and GA_3_-oxidases (GA3OX) exhibited significantly induction in the *MIR408-OX* transgenic plants compared with wild type. On the contrary, transcripts levels of the biosynthetic negative regulators of *PIL5* and *DAG1* were dramatically decreased in the transgenic plants. Expression level of GA2-oxidase (GA2OX) for GA deactivation did not show obvious difference between the wild type and the *MIR408-OX* transgenic plants. Moreover, GA contents were examined in the rosette leaves of wild type and miR408 over-expressing plants grown in soil for 2 weeks. The levels of GA_4_, the major endogenous bioactive GA in Arabidopsis, were significantly increased in the *MIR408-OX* plants although that of GA_9_, the immediate precursor of GA_4_, showed a slight increase (**Figure 3C**). In addition, the contents of GA_1_ as well as its immediate precursor GA_20_ were dramatically increased in the *MIR408-OX* plants. Taken together, these observations suggest that accumulation of miR408 might directly or indirectly modulate the cytoplasmic growth and/or GA biosynthesis to promote cell expansion and thus leading to the larger phenotypes.

### Increased Photosynthesis and Chloroplastic Copper Content by miR408 Overexpression

**FIGURE 4:**
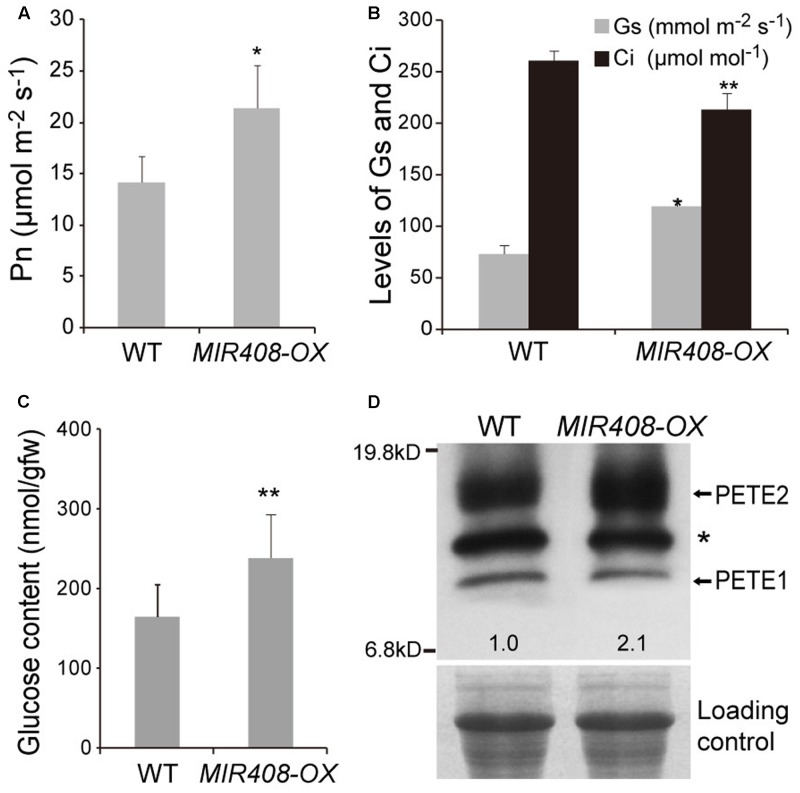
Enhanced photosynthesis by overexpressing *MIR408*. **(A)** Comparison of net photosynthetic rate between the wild type and *MIR408-OX* transgenic plants grown in soil for 2 weeks (*n* = 5). **(B)** The net photosynthetic rate is positively correlated with stomatal conductance (*G*_s_) and negatively correlated with internal CO_2_ concentration (*C*_i_) (*n* = 5). **(C)** Glucose content in the shoots of plants grown in soil for 2 weeks (*n* = 3). **(D)** Immunoblot analysis of PETE (PC) protein levels in the wild type and *MIR408-OX* transgenic plants. Values represent PETE2 levels normalized against the loading control RPT5 using Image J software and set to one for wild type. Data are means ± SD from *n* biological repeats. ^∗^*p* < 0.01, ^∗∗^*p* < 0.05 vs. the wild type, by two-tailed Student’s *t*-test.

A key element to increased growth and reproduction is photosynthesis. To investigate the impact of increased expression of miR408 on photosynthesis, the rates were measured using a portable photosynthesis system (CIRAS-1, PP Systems) in the fifth rosette leaves of wild type and *MIR408-OX* plants grown in soil for 2 weeks. The net photosynthetic rate was significantly increased in the *MIR408-OX* transgenic plants compared to that of wild type (**Figures 4A,B**). Consequently, the contents of glucose were found to be significantly increased in the rosette leaves of the transgenic plants (**Figure 4C**). In the final step of the linear electron transport of photosynthesis, ferredoxin-NADP^+^-oxidoreductase (FNR) catalyzes the reduction of NADP^+^ by ferredoxin (Fd) and provides the reducing power for CO_2_ fixation in the Calvin cycle (Carrillo and Ceccarelli, 2003). FNR is supposed to be one of the limiting factors in photosynthetic electron transport, and the amount of FNR has been shown to correlate with photosynthetic activity (Hajirezaei et al., 2002). We found that the transcript levels of genes for leaf-type FNRs (*ATLFNR1* and *ATLFNR2*) showed significant increase in the *MIR408-OX* plants (Supplementary Figure S2a).

Plastocyanin (PC), which is encoded by two paralogous genes, *PETE1* (less abundant) and *PETE2* (more abundant) is a copper-binding protein that functions as electron carrier in the thylakoid lumen of the chloroplast (Weigel et al., 2003; Abdel-Ghany, 2009; Pesaresi et al., 2009). Previous studies have indicated that miR408 repress several genes encoding for copper-binding proteins with non-photosynthetic usage (Yamasaki et al., 2007; Abdel-Ghany and Pilon, 2008). It is, thus, plausible that high level of miR408 repress non-photosynthesis related copper-binding proteins and increases copper’s availability for proteins such as PC. To address this question, we first measured copper content in the whole seedlings, and found that there were no differences between wild type and the *MIR408-OX* plants in overall copper content. The *MIR408-OX* plants, however, had higher chloroplastic copper than wild type plants (**Table 1**). Consistent with this observation, transcript level of *PAA1* which specifically delivers copper from cytosol to chloroplast (Shikanai et al., 2003; Abdel-Ghany et al., 2005) was significantly increased in the *MIR408-OX* plants (Supplementary Figure S2b). Thus, elevated miR408 promotes the copper allocation to chloroplast from the cytosol. We next compared the PC levels between wild type and the *MIR408-OX* transgenic plants because PC is one of the major destinations of chloroplastic copper (Ramshaw et al., 1973). *PETE2* was significantly increased in the *MIR408-OX* plants at both the mRNA and protein levels, though PETE1 was unchanged (**Figure 4D** and Supplementary Figure S2c). Taken together, these results suggest that over-accumulation of miR408 promotes photosynthesis by modulating the distribution of copper in plant cells.

**Table 1:** Copper content (μg.g^-1^ fresh weight).

Genotype	Total	Chloroplast	Percentage
WT	0.92 ± 0.07	0.27 ± 0.09	29.34%
*MIR408-OX*	1.18 ± 0.06	0.48 ± 0.11^∗^	40.67%


*Values are from three biological repeats, and star represents significant difference compared with the wild type (two-tailed Student’s *t*-test, *p* < 0.05).*

### Transcriptome Changes in *MIR408-OX* Plants

**FIGURE 5:**
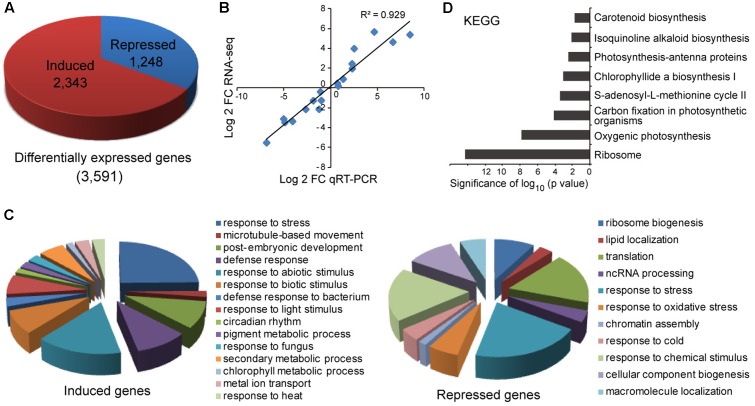
Characterization of genes with altered levels of expression in the *MIR408-OX* transgenic plants. **(A)** Differentially expressed genes between the wild type and *MIR408-OX* plants. **(B)** Correlation between RNA-sequencing and qRT-PCR data on the selected genes. Pearson correlation was calculated using data points representing Log_2_ transformed transcript level ratios of wild type and *MIR408-OX* plants. **(C)** Enriched representative GO terms (*p* < 0.001) in the biological category that associated with induced (left) and repressed (right) genes in the *MIR408-OX* transgenic plants. **(D)** Enriched pathways associated with the differentially expressed genes by KEGG analysis.

To gain more insight into the molecular changes associated with miR408 overexpression, total RNA was extracted from whole seedlings of wild type and the *MIR408-OX* seedlings grown on MS plate for 10 days under continuous light, and subjected to high throughput sequencing. We identified 3,591 differentially expressed genes, of which 2,343 were induced and 1,248 repressed in the *MIR408-OX* transgenic plants, respectively (**Figure 5A** and Supplementary Table S1). To further confirm the RNA sequencing data, we performed RT-qPCR to monitor the transcripts levels on a handful of copper-responsive as well as randomly selected genes. As shown in **Figure 5B**, the results generated by the two methods agreed well. Gene Ontology (GO) analysis revealed that the genes induced in *MIR408-OX* transgenic plants preferentially associated with GO terms such as response to stimulus and stress, microtubule-based movement, chlorophyll metabolic process, pigment biosynthetic process, and metal ion transport (**Figure 5C**). Biological pathways responsive to ribosome biogenesis, lipid location, response to oxidative stress, and RNA processing were greatly enriched among the downregulated genes (**Figure 5C**). Regarding annotated pathways, ribosome, photosynthesis, carbon fixation, pigments biosynthetic processes are among the most significantly enriched terms (**Figure 5D**). Thus, we hypothesize that these transcriptomic changes may contribute to the photosynthesis, myosin-mediated cytoplasmic growth, abiotic stress responses, and better protection of cells against reactive oxygen species (ROS) especially from the higher photosynthesis of the *MIR408-OX* transgenic plants. Overall, these results suggest that overexpression of miR408 influences multiple metabolic processes directly or indirectly and thus impacts on multiple aspects of plant growth and development.

### Effect of miR408 on Reproduction via Modulating Its Target Genes

**FIGURE 6:**
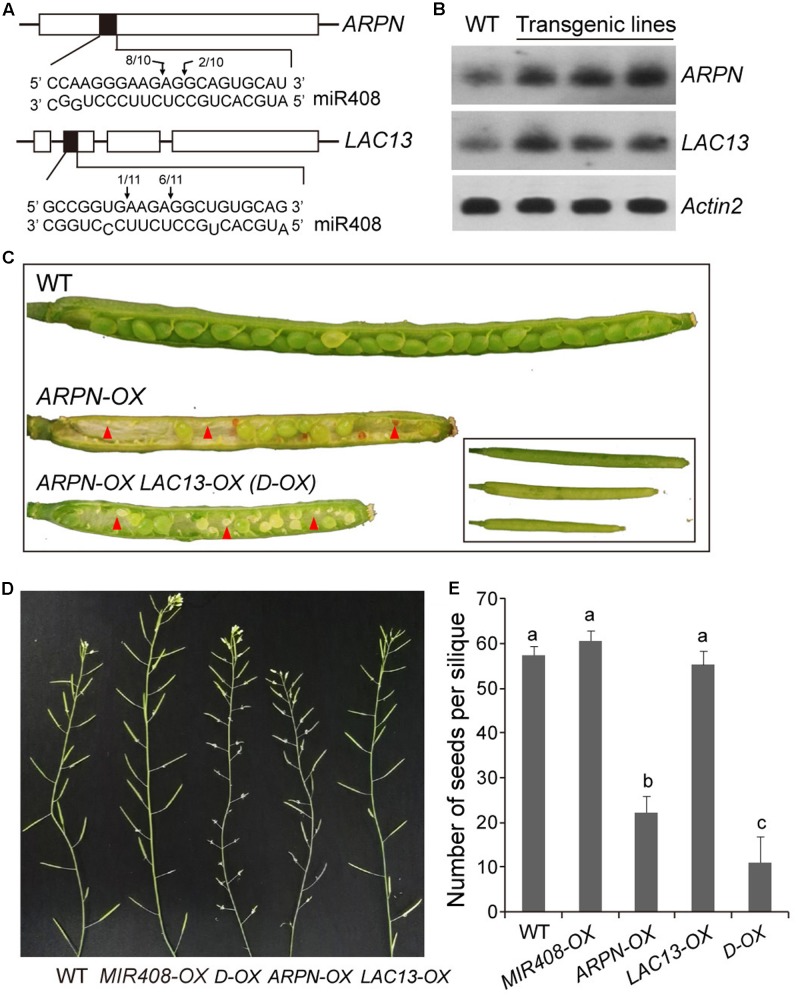
Biomass and seed yield traits of *Plantacyanin* (*ARPN*) and *LAC13* overexpressors. **(A)** Confirmation of miR408 targeting on *ARPN* and *LAC13* by 5′-RACE. The complementary mRNA and miRNA sequences are shown with shaded boxes. Vertical arrows mark the sequenced cleavage sites with the frequency of clones shown. **(B)** RNA gel blot analysis for wild type and *ARPN-OX* or *LAC13-OX* transgenic lines. *Actin2* served as loading control. **(C)** Stereomicroscopy images of siliques obtained from self-pollinated wild type, *ARPN-OX*, and *ARPN-OX/LAC13-OX* (*D-OX*) parental plants. Red arrowheads indicate abnormal ovules. **(D)** Comparison of silique development on 6-week-old plants of the wild type, *MIR408-OX*, *ARPN-OX*, *LAC13-OX*, and *D-OX* genotypes. **(E)** Seeds from different genotypes were collected and quantified (*n* = 15). Data are means ± SD. Genotypes labeled with the same letters have no statistical difference, while different letters denote groups with significant differences (ANOVA, *p* < 0.01).

Identification and functional analysis of the targets of miRNA is crucial for understanding the biological function of miRNA. To further dissect the function of miR408, two of its target genes, *Plastacyanin* (*ARPN*) and *LAC13* (**Figure 6A**), were analyzed. We obtained two T-DNA insertion lines of SALK_091945 for *ARPN* and SALK_023935 for *LAC13* from the Arabidopsis Biological Resource Center (ABRC). Unfortunately, we did not see any visible phenotypes for both mutants under normal growth conditions, which are most likely due to the functional redundancy of the miR408 target genes, especially for those laccase members. Thus, we resorted to the overexpression approach to determine their functions. In order to create *ARPN* or *LAC13* overexpressors, we used the CaMV35S promoter to drive protein expression in Arabidopsis. As shown in **Figure 6B**, homozygous transgenic plants contained higher transcript levels of *ARPN* or *LAC13* compared with the wild type. Compared to wild type, the *ARPN*-OX transgenic lines showed shorter siliques and increased silique number of inflorescence (**Figures 6C,D**). Seed formation was dramatically decreased when siliques were randomly picked for seed counts (**Figure 6E**) and thus led to less seed yield although the fresh and dry weight showed no obvious differences from the wild type (**Table 2**). For the *LAC13-OX* transgenic plants, we did not observe any obvious phenotypic changes at either vegetative or reproductive stage (**Table 2**). However, the F1 progenies of *ARPN-OX* and *LAC13-OX* plants showed much shorter siliques and significantly reduction of seeds number and seed yield although the silique number of inflorescence increased (**Figures 6C–E**). In addition, the F1 progenies exhibited significant reduction of fresh and dry weight compared with the wild type (**Table 2**). Meanwhile, the transgenic plants (*amiR408*) with decreased level of endogenous miR408 generated by the artificial miRNA approach reported in the previous study (Zhang and Li, 2013) showed similar phenotypes to the *ARPN-OX*/*LAC13-OX* plants (Supplementary Figure S3 and **Table 2**), suggesting that miR408 may play a role in control of reproductive development by regulating its target genes. Taken together, these results demonstrate that miR408 appears to have pleotropic effects on plant growth and development.

**Table 2:** Comparison of biomass and seed yield among various genotypic plants.

	WT	*ARPN-OX*	*LAC13-OX*	*D-OX*	*AmiR408*
Fresh weight (g)	0.18 ± 0.06	0.16 ± 0.03	0.19 ± 0.05	0.11 ± 0.05^∗^	0.12 ± 0.03^∗^
Dry weight (mg)	13.4 ± 2.3	12.9 ± 3.6	13.7 ± 2.8	10.2 ± 3.3^∗^	9.8 ± 1.4^∗^
Seed yield (mg)	105.3 ± 16.1	38.7 ± 5.2^∗^	99.6 ± 8.4	14.5 ± 4.2^∗^	23.1 ± 3.6^∗^


*Values are means ± SD from n biological replicates, where *n* = 20 for fresh weight or dry weight, and *n* = 10 for seed yield. Stars represent significant difference compared with the wild type (two-tailed Student’s *t*-test, *p* < 0.05).*

## Discussion

MiR408 is among the most conserved miRNA families and has so far been annotated in more than 30 plant species, suggesting that its role is fundamental to plant development and function (Axtell and Bowman, 2008; Kozomara and Griffiths-Jones, 2011). It has shown in the earlier studies that miR408 is involved in photomorphorgenetic development, copper-light signaling pathway, and biotic stress responses as well as vegetative biomass in Arabidopsis seedlings (Zhang and Li, 2013; Zhang et al., 2014; Ma et al., 2015). In our present work, constitutive expression of miR408 influences various developmental stages, and promotes vigorous plants growth and seed yield by increasing photosynthetic efficiency. Therefore, miR408 likely have pleiotropic effects on plant growth and development.

Transgenic plants overexpressing *MIR408* exhibited vigorous growth phenotypes such as increased leaf area, petiole length, plant height, and biomass yield. Tsukaya et al. (2002) demonstrated that both increased cell number and increased cell length lead to larger size of leaf or petiole length. Our results showed that increased cell size but not cell number leads to the larger phenotype of the *MIR408-OX* transgenic plants. Interestingly, a previous study demonstrated that myosin-mediated cytoplasmic streaming is a key determinant of plant size (Tominaga et al., 2013). In Arabidopsis, there have 13 myosins in the class XI family (Reddy, 2001). Among the nine tested myosin genes, eight of them exhibited significantly increased levels in the *MIR408-OX* transgenic plants compared with wild type (**Figure 3A**), implying that induction of myosin might be an explanation for the larger morphology in the transgenic plants. Apart from copper, expression levels of *MIR408*, as well as its target genes, were investigated in different plant species in response to various environmental conditions, such as light, cold, salinity, oxidative stress, mechanical stress, drought and osmotic stress (Sunkar and Zhu, 2004; Lu et al., 2005; Kantar et al., 2010; Li et al., 2010; Trindade et al., 2010; Sunkar et al., 2012; Zhang et al., 2014; Rajwanshi et al., 2014). These observations may suggest a new adaptation strategy to environmental fluctuations for plants survival such as flood and canopy shade by manipulating miR408 abundance.

Leaf position plays a vital role for photosynthesis, and the control of leaf petiole elongation is an important mechanism to ensure plant leaves at appropriate positions. Several key regulators that control leaf petiole elongation had been identified (Tsukaya et al., 2002). Mutation of the *ROTUNDIFOLIA 3* (*ROT3*) gene encoding a protein of the cytochrome P450 family results in short leaf petioles, and overexpression of the *ROT3* gene in transgenic Arabidopsis produces elongated leaf petioles (Tsuge et al., 1996; Kim et al., 1999). In addition, knock-out of *ACAULIS 2* gene (*ACL2*) suppresses leaf petiole elongation as well as flower stalks (Tsukaya et al., 1995). Recently, three related receptor-like kinases encoded by *HERCULES1* (*HERK1*), *THESEUS1* (*THE1*) and *FERONIA* (*FER*) that regulate the brassinosteroid signaling pathway were reported to control petiole growth, which mutants showed short leaf petioles phenotypes (Guo et al., 2009). Furthermore, previous studies have revealed that cell extensibility regulated by cell-wall loosening proteins is one of important factors controlling cell elongation in plants (Cosgrove, 2000; Ookawara et al., 2005). Consistently, expression levels of *ACL2*, *ROT3*, *HERK1*, and several of cell-wall loosening genes were significantly changed between the *MIR408-OX* and wild type plants (Supplementary Table S1). These observations suggest that overexpression of miR408 indirectly influences the cell elongation genes and thus leads to elongated petiole. Additionally, hormones, such as gibberellins (GAs), are well known regulators of cell elongation in many species, such as Arabidopsis, maize and rice (Luo et al., 2006; Achard et al., 2009; Bolduc and Hake, 2009). In our study, transcript levels of genes for GA biosynthesis were dramatically increased in the *MIR408-OX* transgenic plants (**Figure 2B**), and an increased production of hormone could be expected (**Figure 2C**), implying that GA-enhanced cell elongation could play a role in the larger phenotypes as well.

In addition to miR408, there are several copper-regulated miRNAs that respond to external copper concentrations in Arabidopsis, e.g., miR397, miR398, and miR857, which together repress the levels of mRNA transcripts for a number of copper-containing proteins under Cu-limited conditions (Abdel-Ghany and Pilon, 2008; Yamasaki et al., 2009). Constitutive expression of miR397a or miR397b in rice could promote panicle branching and increase grain size and yield (Zhang and Li, 2013). Furthermore, compared to wild type Arabidopsis, the transgenic plants overexpressing miR397b developed more inflorescence shoots and showed increased silique number and silique length, which resulted in higher seed numbers (Wang et al., 2014). In contrast, transgenic plants overexpressing its target gene, *LAC4*, were severely dwarfed with small rosette leaves, short inflorescence stems, short silique, and less seeds, a completely opposite phenotype to that observed for the *miR397b-OX* plants. Previous studies have indicated that under copper deficient conditions, elevated miR408 level can lead to inhibition of mRNA transcripts for copper-containing proteins, thus promoting the preferential delivery of copper from cytoplasm to chloroplast, which in turn ensures photosynthesis (Weigel et al., 2003; Burkhead et al., 2009). Thus, it is not surprising that constitutive expression of miR408 results in higher accumulation of copper in the chloroplast of the *MIR408-OX* transgenic plants compared with the wild type and obviously increased photosynthetic efficiency (**Table 1** and **Figures 4A,B**). Our data presented here may suggest that overexpression of miR408 results in vigorous growth and enlarged morphology as well as increases biomass and seeds yield through promoting photosynthetic efficiency. In terms of silique size and seeds yield, the *MIR408-OX* transgenic plants are similar to the *miR397b-OX* ones in Arabidopsis. However, the *LAC13-OX* Arabidopsis showed no obvious difference from wild type although both *LAC4* and *LAC13* belong to the laccase family. These observations indicate that regulatory mechanism may be distinctive for individual copper miRNAs.

It should be noted that actual plants’ photosynthetic efficiency is rather low from 0.1 to 8% because of inefficient conversion of solar light to electric energy (Zhu et al., 2010). To overcome this limitation, genetically modify plants can be developed to achieve greater efficiencies and enhanced growth. To data, genetic engineering has been widely used to develop new agricultural crops with stress tolerance against degradation of global environment. However, genetically modified plants with desirable agricultural traits by manipulating related regulatory effectors frequently show growth limitation and yield penalties for the interplay between developmental and stress responsive signaling networks (Cabello et al., 2014). In this regard, it becomes desirable to explore candidate genes that can confer stress tolerance without restricting the plant growth and yield. Our results demonstrate that the transgenic plants overexpressing *MIR408* showed significantly increased photosynthetic rates and produced higher biomass and seed yield. More recently, Ma et al. (2015) reported that overexpression of miR408 led to improved tolerance to abiotic stresses in Arabidopsis, such as salinity, cold, and oxidative stress. Together with a previous study that overexpression of miR408 significantly increased drought tolerance in chickpea (Hajyzadeh et al., 2015), it is plausible that the *MIR408-OX* transgenic plants could be resistant to stress conditions. Thus, miR408 may provide an important cross-link between plant growth, development and stress response, and play a central role in plant survival. Furthermore, it is well known that miR408 targets three laccase genes, *LAC3*, *LAC12*, and *LAC13*, which polymerize monolignols into lignin (Bao et al., 1993; Mayer and Staples, 2002). Thus, a reduction of lignin deposition could be expected in the *MIR408-OX* plants as well. This information may have potential to improve a wide range of plant species for use as bioenergy feedstocks. Taken together, our results suggest that *MIR408* can be of potential interest as a candidate gene in developing new agricultural crops. Further exploration of miR408 in various plant species including crops will thus provide much needed insight as to the coordinated control of the superior phenotypes.

## Accession Numbers

Sequence data from this article can be found in the Arabidopsis Genome Initiative or GenBank/EMBL databases under the following accession numbers: *MIR408* (At2g47015), *Plantacyanin* (At2g02850), *LAC13* (At5g07130), *PETE1* (At1g76100), *PETE2* (At1g20340), *Myosin XI-B* (At1g04160), *Myosin XI-1* (At1g17580), *Myosin XI-1* (At5g43900), *Myosin XI-G* (At2g20290), *Myosin XI-F* (At2g31900), *Myosin XI-J* (At3g58160), *Myosin XI-H* (At4g28710), *Myosin XI-I* (At4g33200), *Myosin XI-K* (At5g20490), *ATGA3OX3* (At4g21690), *ATGA20OX2* (At5g51810), *ATGA20OX4* (At1g60980), *ATGA20OX1* (At4g25420), *ATGA20OX3* (At5g07200), *ATGA2OX1* (At1g78440), *ATGA2OX6* (At1g02400), *DAG1* (At3g61850), *PIL5* (At2g20180), *LFNR1* (At5g66190), *LFNR2* (At1g20020), *RFNR1* (At4g05390), *RFNR2* (At1g30510), *HMA1* (At4g37270), *PAA1*(At5g35590), *PAA2* (At5g21930), *ACTIN2* (At3g18780). The T-DNA insertion lines are SALK_091945 for *Plantacyanin* and SALK_023935 for *LAC13.*

## Author Contributions

Project design: ZS, YW, and HZhang. Data cultivation and collection: ZS, YW, LZ, SL, HZhang, HL, and HZhao. Data analysis: ZS, YW, LZ, SL, and HZhang. Writing: ZS and HZhang.

## Conflict of Interest Statement

The authors declare that the research was conducted in the absence of any commercial or financial relationships that could be construed as a potential conflict of interest.

## Footnotes

1 https://www.neb.com
