# Supplementary material for: Constitutive Expression of miR408 Improves Biomass and Seed Yield in Arabidopsis
Source: Front Plant Sci. 2018 Jan 25;8:2114. doi: 10.3389/fpls.2017.02114 (PMC5789609; doi:10.3389/fpls.2017.02114)
Supplement: Supplementary file 1 [file Table_2.DOCX]

**Supplemental Table 2.** Oligonucleotide sequences for the primers used in this study

|  | **Forward primer (5’ to3’ )** | **Reverse primer(5’ to 3’)** |
| --- | --- | --- |
| **For RT-qPCR** | | |
| *ATGA3OX3* | CGATCTCCCG ACGAGTCCAC | TCATCGGAATAGAAGAAGCG |
| *ATGA20OX2* | CATGGCGTCA GCGAGTCACT | CAAACTGCTC GAACTCTTGT CC |
| *ATGA20OX4* | TTCCAATCCT CCAAGTCCCT G | GGTTTCACCC ACCTTCCTCT G |
| *ATGA20OX1* | ATCACGGCAT CAGCGAGGAG | GCATCGCAGA AGTAATCTTG AACG |
| *ATGA20OX3* | CAGCACTCGC ACCACATACC T | CCCACTTCCT CTGAGCCTTC T |
| *ATGA2OX1* | TGGAGAAGAT CACAGATGGG CTA | GCTAAGAGGA CATGGTGGAT AGTG |
| *ATGA2OX6* | AAGCCTGTGA GGTTAATGGG TT | CTGTCGGATT TGCGTGGAGA |
| *DAG1* | TAACGTCCCA GTCGGAGGTA GC | TGGCTTGAGA AAAGAATCGG TG |
| *PIL5* | TCTATGGATCCTCAGCAGCAAC | GAAATTCATGAAGTTCCTCAC |
| *XI-B* | CCGTTTCAAGCAACAATTGGT | TGCTTCCTAGTAGGATAACCAG |
| *XI-1* | ATGGCTGCTCCAGTCATAATTG | TTCGTAAGACTCCAGGCTCATG |
| *XI-G* | GCTTTCATGGCTTGTTCTACAG | ACTCCTGGTTCATGTAAATACG |
| *XI-F* | ATAATGCGGCTTCAAGACGT | CGTATGCTGCTAAGTGTTGCTTG |
| *XI-J* | AGGAAATATTCTTATTGCCGTCA | AGTATGCAAGGTAACGCATGAGC |
| *XI-H* | TCTCAAGCTTCCATTGAAATC | ACATTGACTGTAGTACAAGCCA |
| *XI-I* | TCAATTTCATTATCTAAATCAAAGC | AAGATCAGCAGCCATCTGCAGATG |
| *XI-K* | GCGTTTACAACTAGAAAAGCGA | AGCTTCCACCTCTGATGTTAATG |
| *XI-2* | GTTCCTAGATGCAGCCAAGTCA | AACTTCACCATCAATCCATGC |
| *ARPN* | GAGGCAGTGCATCATGGTCG | GAGGTCCGTTTGAATCTTCCA |
| *LAC13* | TTCACTTGTCAATGCAGAAGTTCAC | TCTCATTATCCGCCCTCCGCTCTC |
| *CCS* | ATCAATTCTCAGGTCAGTGG | GCTCGATCCTCCTGATGGAGA |
| *CSD1* | GCCTGGTCTTCATGGTTTCC | TGAGTTCATGGCCTCCCTTTC |
| *CSD2* | AACAACATGACACACGGAGC | CCAATCACACCACATGCCAA |
| *FRO4* | TTGCTCTTTTGGCTTGGTCC | AAGCCCTAAACTTTGCCTGC |
| *YSL2* | CCATCTTACGCCAAACCTCG | TCAGCTCTTCCTTCACCTGG |
| *COPT2* | TCACCGATCCTACGTGTCAG | CTGGTCATCGGAGGGTTTCT |
| *PETE1* | TAAAGCTCGCCGTCAGCTCAA | CTACACGAACAAGAGAATATCTAG |
| *PETE2* | CTCATAAACTCGATCGAACCAAAGC | CTAAATACCTCTATGAAAAGCGCT |
| *HMA1* | AACTGGTGAGATTGTGCCTGTAGA | TATTAGAATGTGCTTCCTCGGTCA |
| *PAA1* | CATCATTAGGAGCAAGAAGAGGATTAC | GACAATAGCAACAGCACCAGACC |
| *PAA2* | TTGTGCTCCTTGGTCGTTCTTTG | TCCCAGTTAATTGTTCCCGCTGA |
| *LFNR1* | CTCTATCGCGAGTAGTGCCA | CTTCATCACCCGGCTTCAAG |
| *LFNR2* | GCAGAGAACAAGCGAACGAT | TCAACGTTCCATTGCTCTGC |
| *RFNR1* | TGTTGTCTCTTCTGGGCCTT | AGTGCAACGGACATCTGAGA |
| *RFNR2* | CATACAGAGG CTACTTACGT CGAAT | ATCTTTCCAC CTTTCTTGTT CTTCT |
| *Actin2* | ATTACCCGATGGGCAAGTCA | CACAAACGAGGGCTGGAACA |
| **For plastmids construction** |  |  |
| *35S:ARPN* | GGACTAGTTTGTACATTTACTCTTCAATTCATCC (*Spe*I) | ACGAGCTCCGTTTGAATCTTCCAAACTACAAAACC (*Sac*I) |
| *35S:LAC13* | GCTCTAGAATGGAGCAACTTCGACCCTTC (*Xba*I) | GCGAGCTCTTTTGTGAAACAAATTTGAGAATATACAAG (*Sac*I) |
