# Supplementary figures and images for: Constitutive Expression of miR408 Improves Biomass and Seed Yield in Arabidopsis
Source: Front Plant Sci. 2018 Jan 25;8:2114. doi: 10.3389/fpls.2017.02114 (PMC5789609; doi:10.3389/fpls.2017.02114)

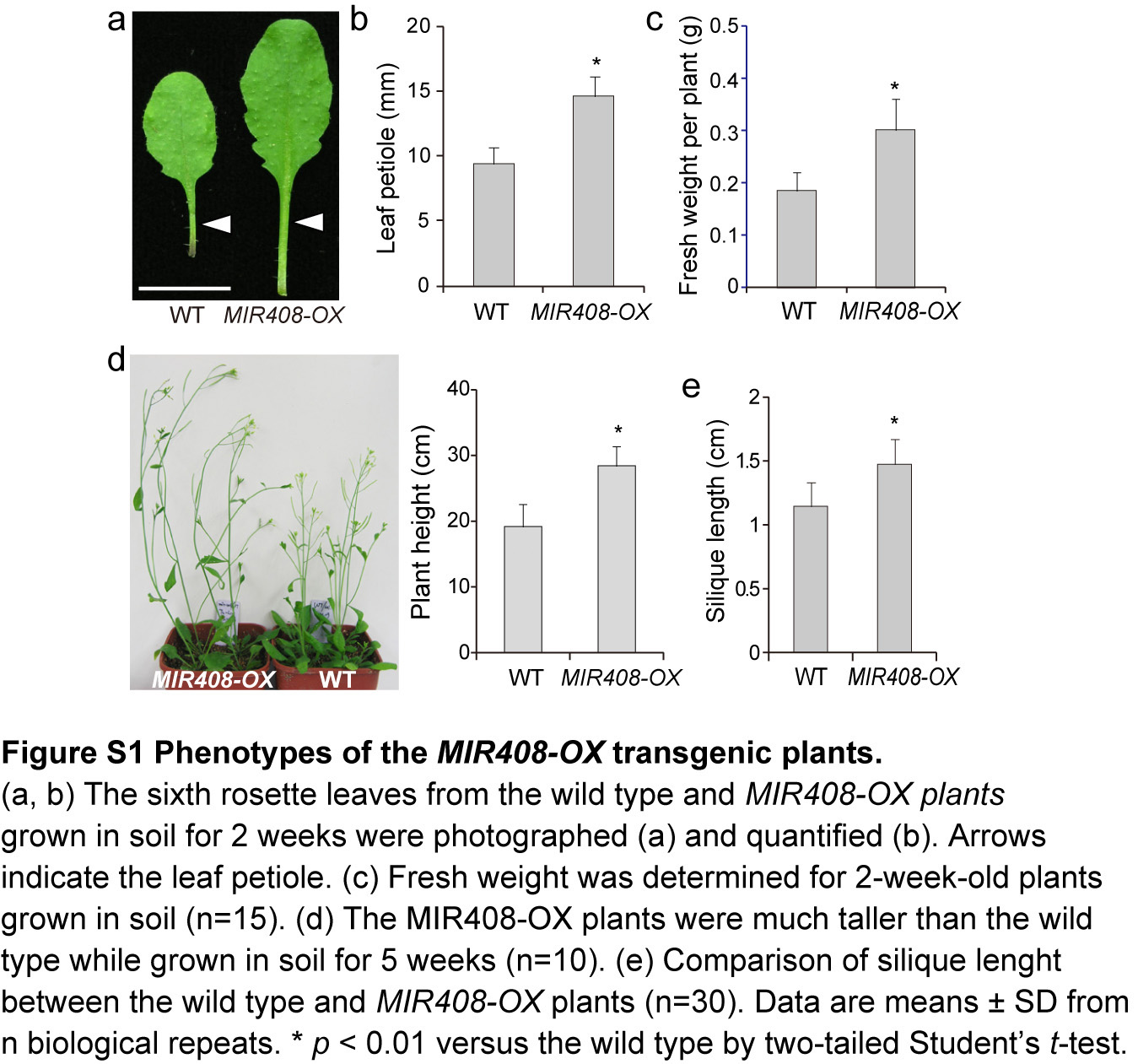

Supplement: Supplementary file 4 [file Image_1.JPEG]

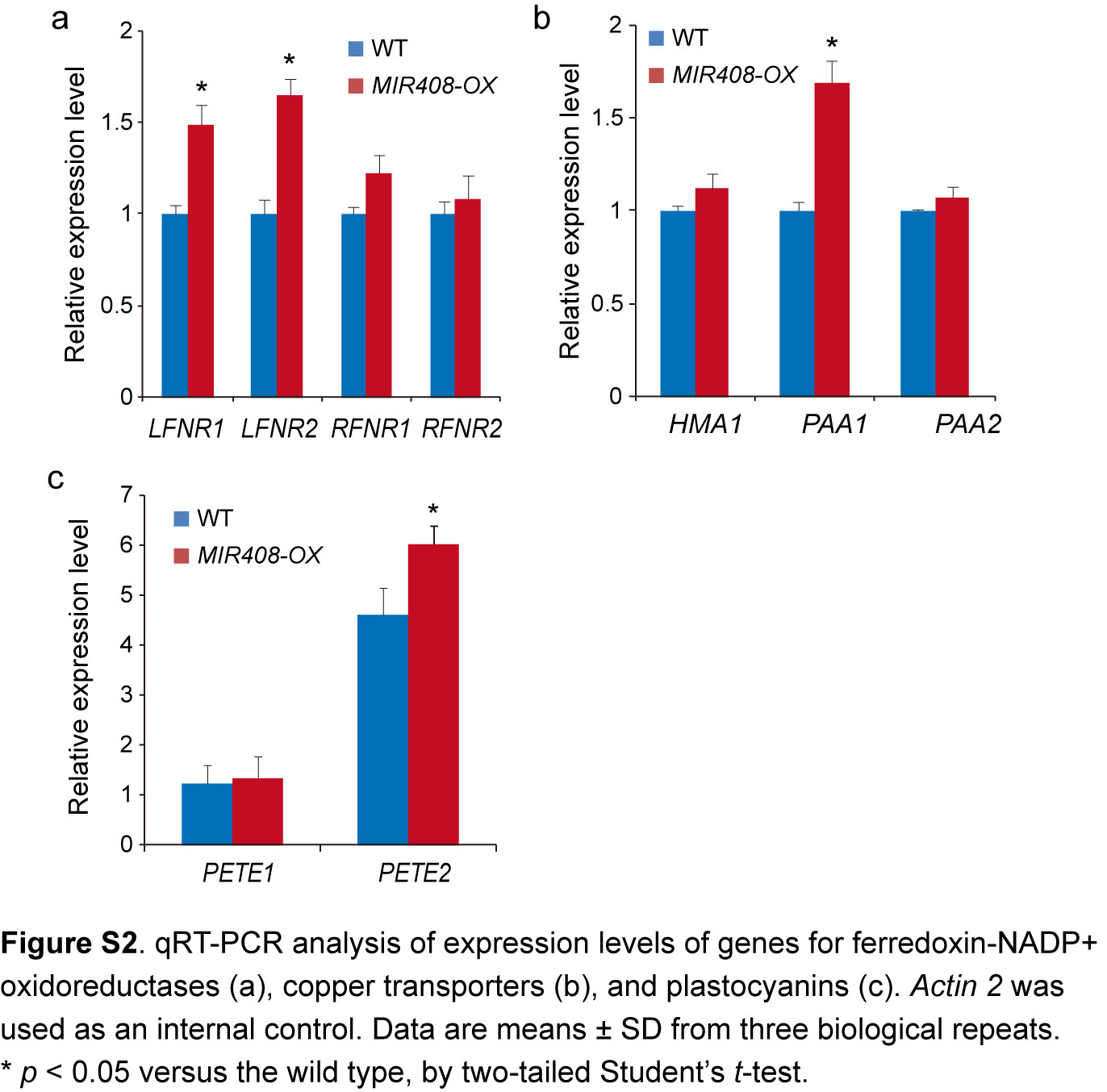

Supplement: Supplementary file 5 [file Image_2.JPEG]

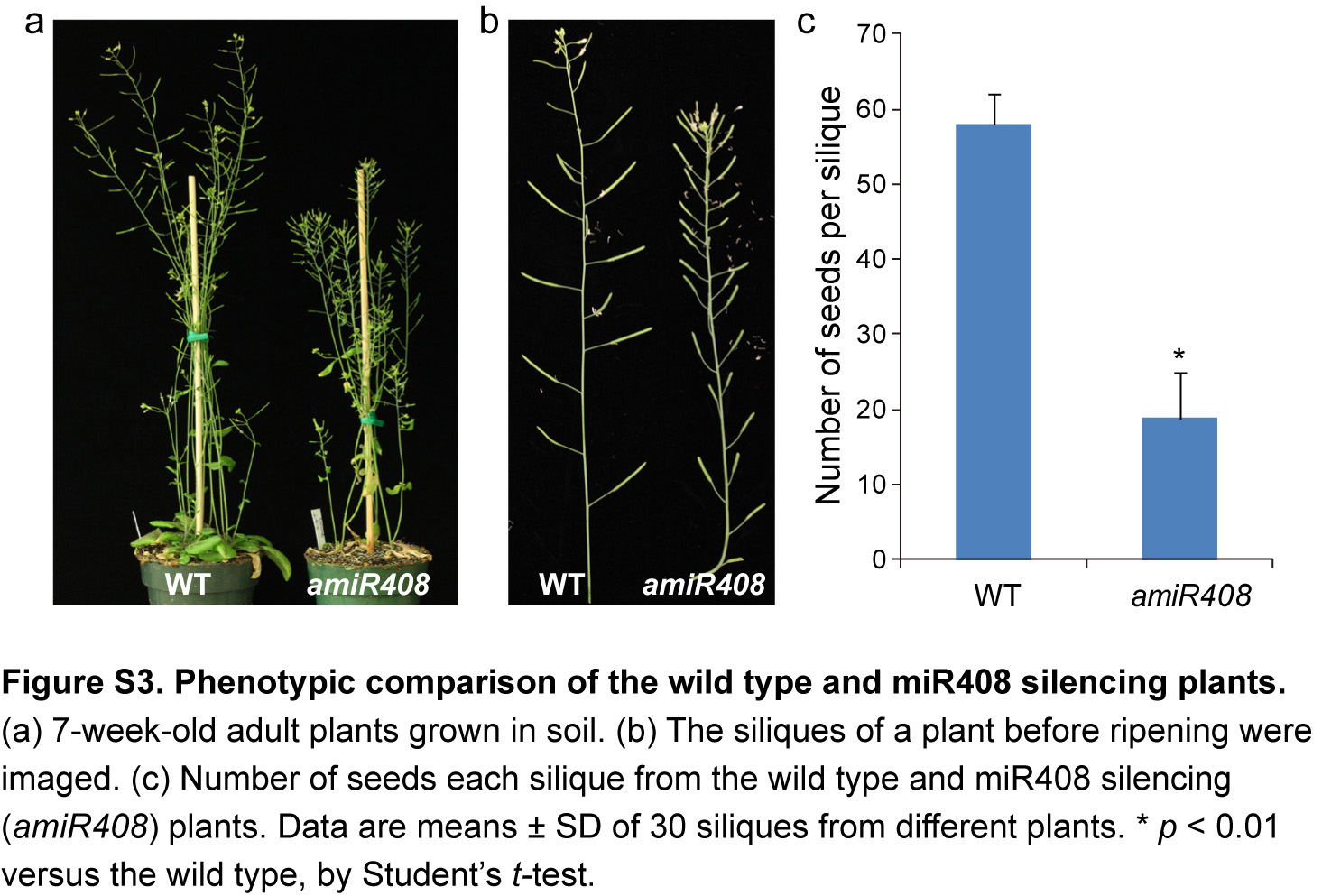

Supplement: Supplementary file 6 [file Image_3.JPEG]
